# 3D printed vitamin D impregnated catheters for magnetic resonance-guided interventions: proof of concept and imaging characteristics

**DOI:** 10.1186/s41205-025-00273-y

**Published:** 2025-06-13

**Authors:** Liam O. Cunningham, Aravinda Ganapathy, Cihat Eldeniz, Jeffery A. Weisman, Kevin E. Lindsay, Udayabhanu Jammalamadaka, Karthik Tappa, Amber Salter, Hongyu An, Pamela K. Woodard, David H. Ballard

**Affiliations:** 1 School of Medicine, Washington University in St. Louis, St. Louis, MO USA; 2 Mallinckrodt Institute of Radiology, Washington University School of Medicine, 510 S. Kingshighway Blvd, Campus Stop 8131, St. Louis, MO 63310 USA; 3 Department of Biostatistics, Washington University School of Medicine, St. Louis, MO USA

**Keywords:** 3D printing, MR-guided interventions, Catheter tracking, MR-compatible materials, Custom medical devices

## Abstract

**Background:**

Catheters used for magnetic resonance (MR)-guided interventions require intra-catheter coils and often produce artifacts. This study aimed to fabricate 3D-printed catheters impregnated with vitamin D solution to allow for optimal visualization during MR-guided procedures.

**Methods:**

3D printing was used to fabricate catheters impregnated with vitamin D solution. Computer-aided design files were generated for a size 18 French catheter prototype with a compartment for vitamin D solution to be manually introduced into the catheter’s lumen and sealed via thermoplastic welding. Polylactic acid (PLA) bioplastic was 3D printed into filaments via material extrusion (FDM^®^, Stratasys, Eden Prairie, MN) on a 5th generation Replicator 3D printer (MakerBot). Three different forms of vitamin D were used, cholecalciferol, ergocalciferol, and calcitriol, and 0.9% normal saline served as a control. Three prints of each catheter type were fabricated and scanned using a 1.5 T MR whole body scanner (Avanto, Siemens Healthcare) inside a small flex loop surface radiofrequency (RF) coil. A 3D gradient recalled echo (GRE) sequence was used with the following acquisition parameters: 4.52/11 ms TE/TR, 15° flip angle, 256 × 256 matrix with 0.5 mm × 0.5 mm in-plane resolution, 24 coronal slabs, 2 mm thickness, and 140 Hz receiver bandwidth. Three averages were used to improve the signal-to-noise ratio (SNR). The GRE sequence was run with 4 different flip angles: 3°, 15°, 30°, and 45° to perform T1 mapping.

**Results:**

All 3D-printed catheters impregnated with vitamin D produced a signal on MR. SNR for vitamin D catheters was similar across the various forms of vitamin D: mean SNRs for 100% cholecalciferol, ergocalciferol, and calcitriol were 138, 139, and 130. Mean SNR and contrast-to-noise ratio (CNR) for vitamin D catheters were significantly higher than the control saline catheter (*p* < 0.001, for both SNR and CNR). T1 values were lower in vitamin D-impregnated catheters compared to the saline control (228 ± 67 ms and 3371 ± 493 ms, respectively; *p* < 0.0001), indicating a better signal.

**Conclusions:**

3D printing of catheters impregnated with vitamin D is feasible and can potentially optimize MR-guided procedures.

## Background

Vascular and non-vascular image-guided interventions are primarily performed with fluoroscopic, ultrasound, or computed tomography (CT) guidance for real-time or intermittent visualization over the course of a procedure. Despite improved soft tissue contrast and spatial resolution over other imaging modalities, magnetic resonance (MR), has yet to be widely incorporated into the interventionalist repertoire/toolkit, in large part due to unfamiliarity, lack of precedence, availability, ferromagnetic restrictions in the types of materials that can be used in an MR suite, and visibility of procedural instruments, such as catheters [1–3]. Currently, there are no commercially available MR-optimized catheters.

Currently, MR-guided interventions are used in a limited set of clinical applications, such as MR-guided breast biopsies and arthrograms, but have the potential to be utilized in far more complex procedures, taking advantage of MR imaging properties. Potential applications include cryo- or thermal-ablative therapies for neoplastic lesions of the hepatobiliary, musculoskeletal, and genitourinary systems, where MR thermometry offers improved real-time monitoring of the ablation zone [3, 4]. Additionally, the superior spatial resolution of MR allows for increased precision and targeting of small/difficult lesions for biopsy or local therapy over current methods [3].

Traditional procedure catheter materials include polyether block amide, polyurethane, polyethylene terephthalate, and polyimides, none of which are documented to be visible under MR [5]. To address this issue, two primary approaches are utilized as workarounds: ‘passive’ tracking uses metallic markers or contrast to tag/label the catheters, and ‘active’ tracking, which involves attached radiofrequency coils or antennae [6, 7]. These methods have allowed for in vivo catheter monitoring during MR but are not without drawbacks. Notably, due to the addition of the coil in active tracking, there is concern for the heating of surrounding tissues, as well as a compromise of the flexibility of the catheter [8, 9]. Passive tracking is complicated by difficulty differentiating catheters from background tissues (contrast) and the resultant need for full image reconstruction to adequately visualize the catheter [2, 10].

The rapid development and growth of 3D printing technology and material science in the past decade offer the ability to manufacture custom medical instruments that can improve the visibility and functionality of instruments for MR-guided procedures [11, 12]. Through impregnating 3D-printed catheters with vitamin D solutions, this study explores the feasibility of this technology to enhance visibility during MR-guided catheter-based procedures [13].

Previous research has highlighted the potential of 3D printing and materials as it pertains to MR visibility, particularly in the use of MR-visible phantoms. Of note, Mitsouras et al. explored the possibility of using 3D printing to generate MR-visible cervical spine phantoms, through which they identified Objet High Temperature RGD525 (Stratays) as an MR-visible material option, which is comprised of 2-propenoic acid, 1,7,7 trimethylbicyclo[2.2.1]hept-2-yl-ester or isobornyl acrylate [14]. Similar acrylic photopolymers are present in the literature which have shown promise in printing phantoms for use in pre-procedural simulation [15, 16]. However, when considering the use of these materials in intraprocedural devices such as catheters in vivo, which require a high level of resolution in real-time, the issue of chemical shift artifacts arise at the material interfaces, an issue seen with these types of polymers [13].

The primary objective of this study is to develop and evaluate 3D-printed catheters impregnated with vitamin D solution to optimize visualization during MR imaging. By addressing the challenges currently faced by MR-compatible interventional devices, this study seeks to advance the application of MR-guided interventions in clinical practice, enhancing the accuracy and safety of interventions.

## Methods

### Design

Institutional review board approval was not required for this in vitro study. Materials were fabricated in a dedicated 3D-printing laboratory, and constructs were imaged in an imaging research facility. 3D printing was used to construct catheters with vitamin D solution embedded in their tips.

**Fig. 1:**
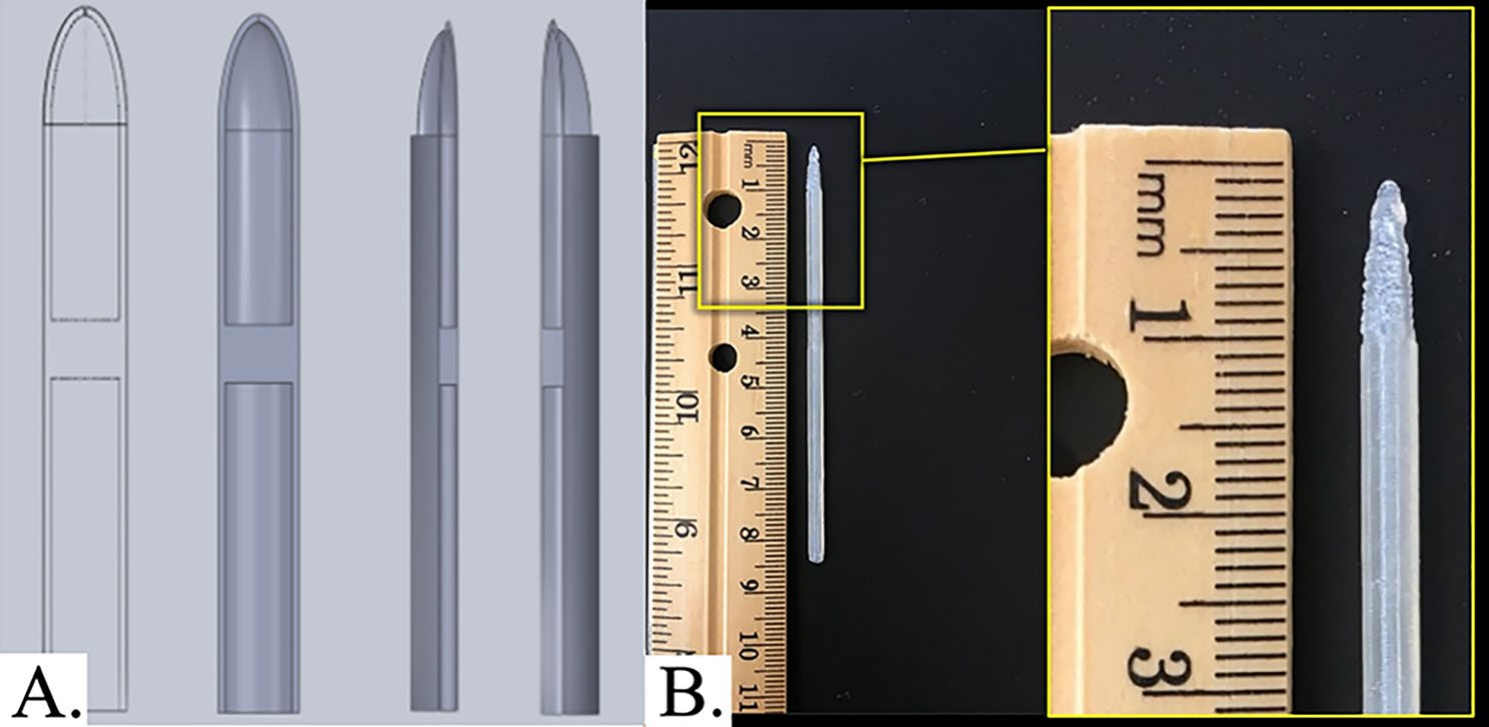
Catheter model with a compartment in its tip for vitamin D solution with a volume of 300 µL. (**A**) Schematic model views; (**B**) Photograph of the 3D-printed catheter alongside a standard ruler. The inset shows a magnified view of the tip region to demonstrate the external geometry and printed dimensions

## Modeling and 3D printing

Catheter geometry was designed in SolidWorks (Dassault Systèmes), with a focus on internal channel architecture to accommodate vitamin D solution. The CAD models were exported as STL files and sliced using MakerBot Print software. Design parameters included catheter length, outer diameter (18 French), and an internal chamber volume of ~ 300 µL integrated into the distal tip. The novel catheter tip design was fabricated using polylactic acid (PLA) bioplastic via material extrusion (FDM^®^, Stratasys, Eden Prairie, MN), in accordance with ISO/ASTM 52,900 standardized terminology for additive manufacturing, using a 5th Generation Replicator 3D printer (MakerBot) [17]. Each catheter was printed with an embedded internal reservoir (~ 300 µL) designed in the CAD model and fabricated as a sealed cavity during printing. Following fabrication, vitamin D solutions were manually introduced into the compartment through a fill port and sealed using a thermoplastic welding technique to minimize leakage. Computer-aided design files were generated using Solidworks (Dassault Systèmes) for a size 18 French catheter prototype with a compartment embedded in the catheter’s lumen for vitamin D solution (Fig. 1). Three different forms of vitamin D were used: cholecalciferol, ergocalciferol, and calcitriol (Strides Arcolab Limited, India) with 0.9% normal saline (G-Biosciences, USA) serving as the control. These different forms of vitamin D were selected due to slight differences in chemical structure contributing to possible differences in visibility, but similar properties otherwise.

Additionally, a commercial plastic previously described as MR-visible (Objet High-Temperature RGD525, Stratasys) was printed and positioned adjacent to a small saline phantom, which was used to localize the signal for MR parameters. Catheters were placed both on the side and top of these saline phantoms.

## Image acquisition

**Fig. 2:**
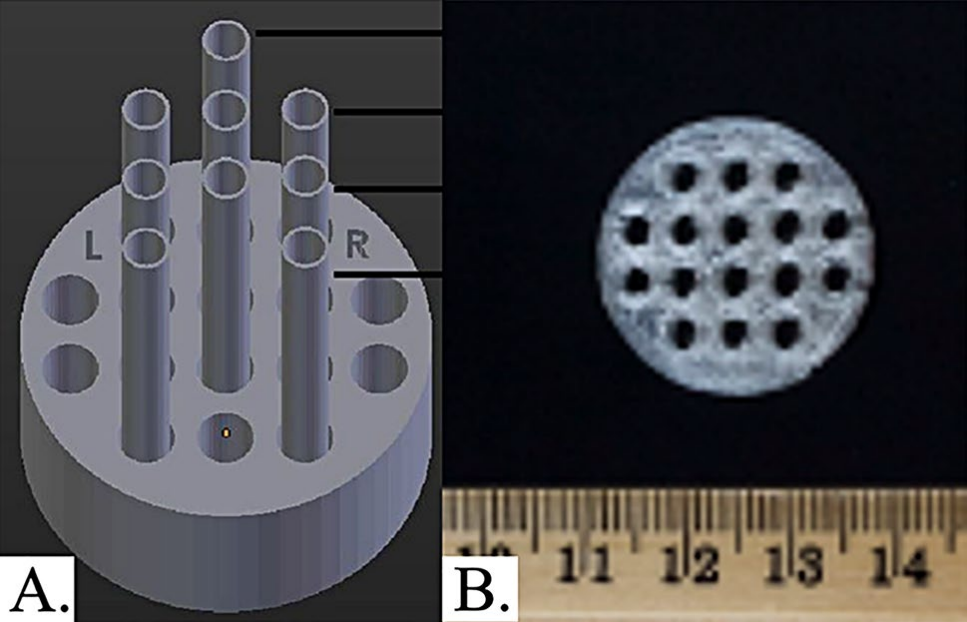
3D printed cylinder to hold catheters in place and image up to 9 simultaneously: (**A**) Schematic and (**B**) photo of 3D printed construct

Three prints of each catheter type were constructed and scanned using a 1.5 T MR whole body scanner (Avanto, Siemens Healthcare) by placing them vertically (Fig. 2) inside a small flex loop surface RF coil. A 3D gradient recalled echo (GRE) sequence was used: 4.52 ms time to echo (TE), 11 ms repetition time (TR), 15° flip angle (FA), 256 × 256 matrix with 0.5 mm × 0.5 mm in-plane resolution, 24 coronal slabs, 2 mm thickness, and 140 Hz receiver bandwidth. Three averages were used to improve the signal-to-noise ratio (SNR) and contrast-to-noise ratio (CNR). The GRE sequence was run with four different flip angles: 3°, 15°, 30°, and 45° to perform longitudinal relaxation time (T1) mapping.

For visualizing the 3D-printed mesh constructed with RGD525, axial and coronal sequences of each were performed initially using a 1.5 T MR whole body scanner (Avanto, Siemens Healthcare). This was followed by scanning with a 3.0 T MR whole body scanner (MAGNETOM Vida, Siemens Healthcare) using both T1- and T2-weighted sequences, including the TR and TE parameters previously described for RGD525 [14].

### Statistical analysis

Descriptive statistics (mean and standard deviation) were used to summarize the SNR and CNR of each isomer in the two different concentrations. Differences in mean SNR and CNR (averaged over all four flip angles) for isomer concentration were examined using scatter plots, box plots, and nonparametric comparisons using the Dunn method, with saline as the control group in MATLAB 2016a (MathWorks, Natick, MA). Additional nonparametric comparisons were performed using the Mann–Whitney test in Prism 10 (GraphPad Software, Boston, MA) to assess T1 values across vitamin D isomer solution concentrations (100%, *n* = 32 and 50%, *n* = 32) compared to saline, *n* = 36.

## Results

**Fig. 3:**
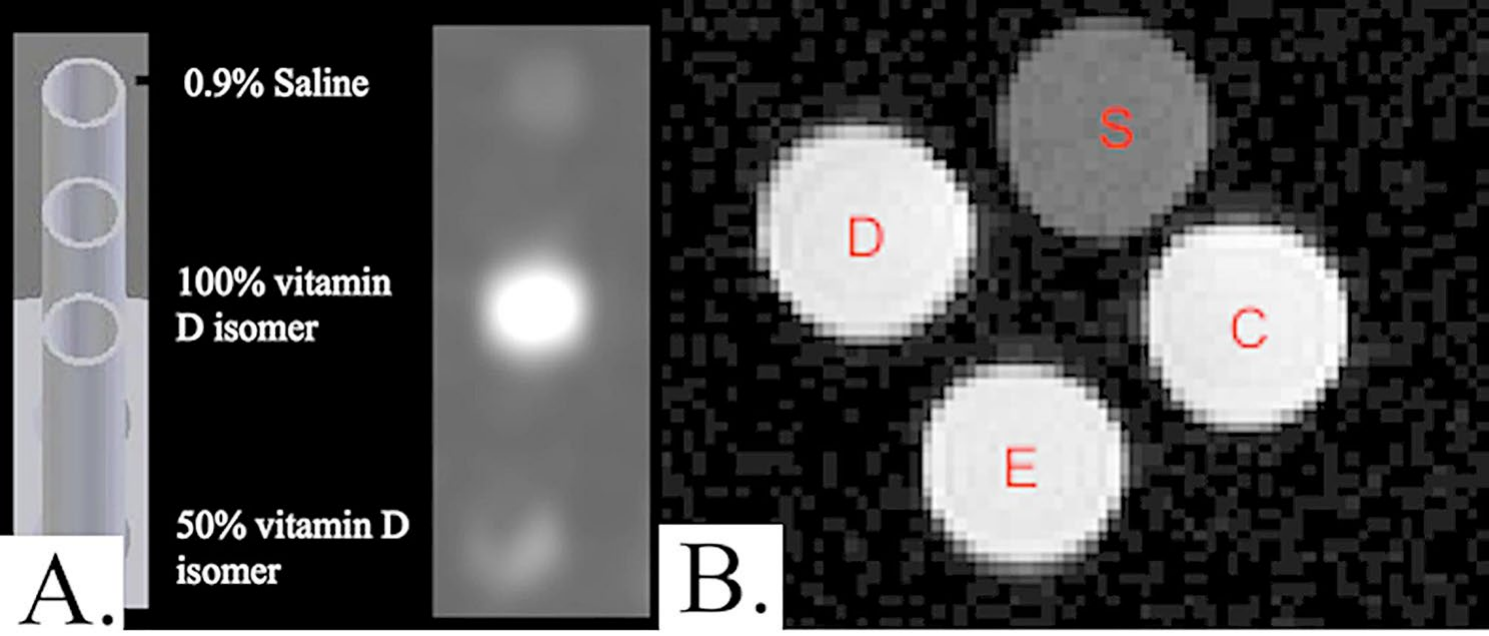
(**A**) MR acquisition showing saline, undiluted, and diluted vitamin D relative to positioning on the 3D printed cylinder and (**B**) Visual comparison MR images of saline (S) to the three vitamin D isomers 100% solutions- D: Cholecalciferol; E: Ergocalciferol; C: Calcitriol

### Visualization of catheters

All 3D-printed catheters impregnated with vitamin D produced a signal on MR (Fig. 3). In contrast, RGD525 catheters did not produce a signal on MR; the saline phantoms showed structure but lacked MR signal enhancement.

## Signal-to-Noise ratio (SNR) and Contrast-to-Noise ratio (CNR)

**Table 1:** Signal-to-noise ratio (SNR) and Contrast-to-Noise ratio (CNR) for various vitamin D solutions (A) 100% concentrations [Cholecalciferol (*n* = 12), Ergocalciferol (*n* = 8), and Calcitriol (*n* = 12)] and (B) 50% [Cholecalciferol (*n* = 8), Ergocalciferol (*n* = 12), and Calcitriol (*n* = 12)], compared to 0.9% Saline (*n* = 36)

**A**	**Cholecalciferol 100**% **(*n*** = **12)**	**Ergocalciferol 100**% **(*n*** = **8)**	**Calcitriol 100**% **(*n*** = **12)**	**Saline(*n*** = **36)**	***p*** **value (vit D vs. saline)**
SNR (mean)	138	139	130	30	< 0.001 (all comparisons)
CNR (mean)	135	137	128	28	< 0.001 (all comparisons)
**B**	**Cholecalciferol 50**% (***n*** = **8)**	**Ergocalciferol 50**% (***n*** = **12)**	**Calcitriol 50**% (***n*** = **12)**	**Saline (*n*** = **36)**	***p*** **value (vit D vs. saline)**
SNR (mean)	42	23	65	30	(0.17, 0.35, 0.13)
CNR (mean)	39	23	63	28	(0.17, 0.51, 0.13)

**Fig. 4:**
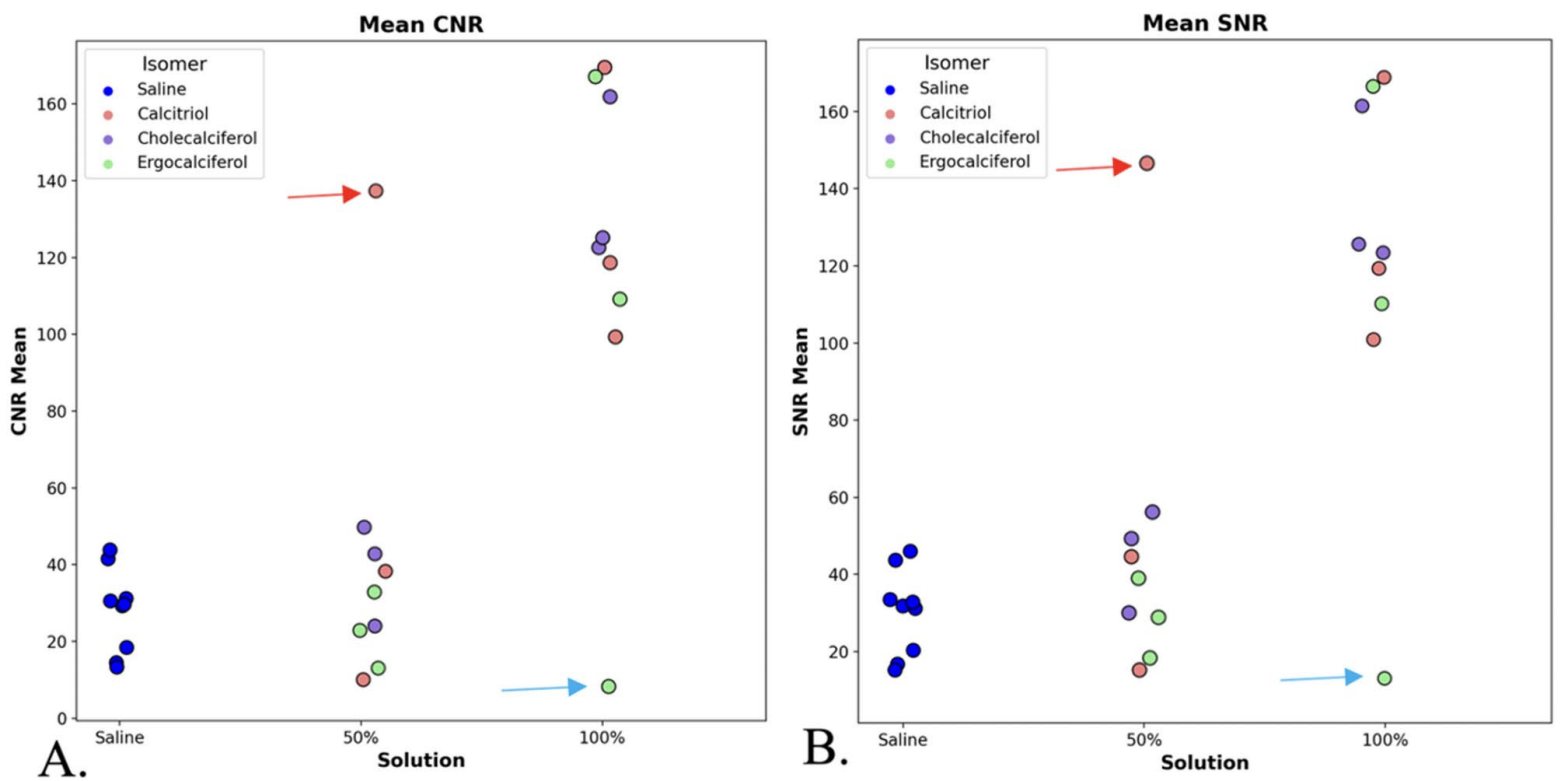
Scatter diagrams plotting the contrast-to-noise ratios (CNRs) (**A**) and signal-to-noise ratios (SNRs) (**B**) of the 3D printed catheters containing saline (control) and Vitamin D isomers (experimental). 100% Vitamin isomers had significantly higher SNR and CNR compared to both saline (control) and the diluted 50% Vitamin D catheters. Two erroneous outliers were not included in the analysis. For one 100% ergocalciferol reading, the catheter leaked and was subsequently poorly imaged (blue arrow), and for another 50% calcitriol catheter with SNR/CNR above 125 was also deemed an outlier (red arrow) due to likely contamination from leakage

**Fig. 5:**
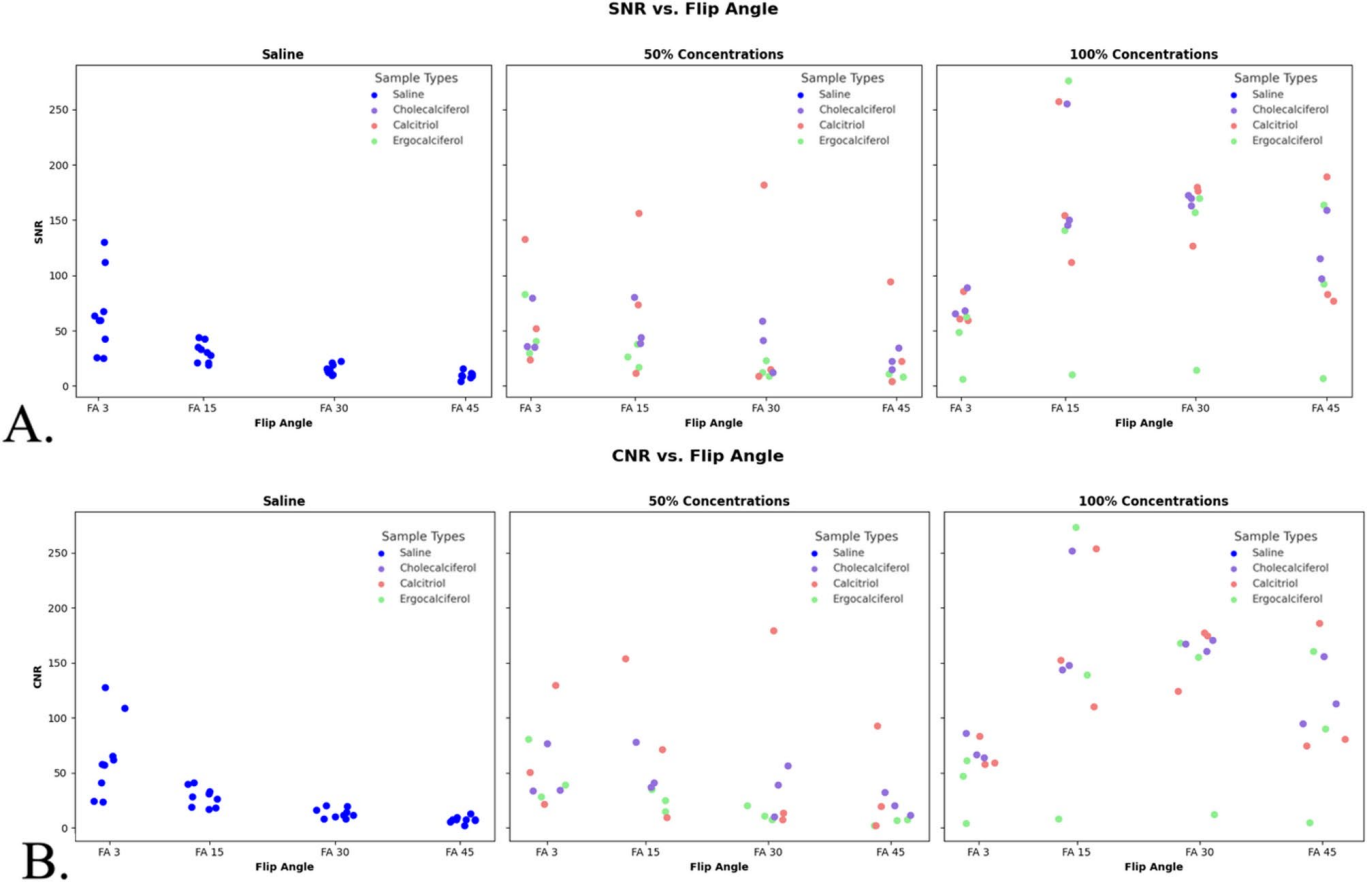
(**A**) Scatter plots showing the Signal-to-Noise (SNR) as a function of flip angle for Saline and Cholecalciferol, Calcitriol, Ergocalciferol, and (**B**) Scatter plots showing the Contrast-to-Noise (CNR) as a function of flip angle for Saline and Cholecalciferol, Calcitriol, Ergocalciferol

When comparing the CNR and SNR data of the three different 100% solutions to one another, via the Kruskal-Wallis test, there was no significant difference (SNR, *p* = 0.8825 and CNR, *p* = 0.8825). The mean SNR for 100% cholecalciferol, ergocalciferol, and calcitriol were 138, 139, and 130, respectively, while the mean CNR was 135, 137, and 128 (Table 1). As such, both SNR and CNR were much higher than the control saline catheters (with SNR and CNR values of 30 and 28, respectively). There is a significant difference in both SNR and CNR between saline and 100% solutions (SNR and CNR both *p* < 0.001), but not between saline and 50% solutions (SNR, *p* = 0.17, 0.35, 0.13 and CNR, *p* = 0.17, 0.51, 0.13) (Fig. 4 and Fig. 5).

## Effect of flip angle

Differences in SNR were identified between FA3 compared to FA30 (*p* = 0.0034) and FA45 (*p* < 0.0001) and between FA15 and FA45 (*p* = 0.0029) for Saline. Differences in SNR were identified between FA3 compared to FA 45 (*p* = 0.0395) for the Ergocalciferol 50% solution (Table 2).

**Table 2:** (A) mean Signal-to-noise ratio (SNR) and (B) Contrast-to-Noise ratio (CNR) at flip angles of 3, 15, 30, and 45 100% concentrations (*n* = 9) and 50% (*n* = 9) compared to 0.9% saline (*n* = 9)

**A**	**Saline (*n*** = **9)**	**50**% **Vitamin D (*n*** = **9)**	**100 Vitamin D (*n*** = **9)**
FA 3	65.14 ± 35.57	56.99 ± 35.26	60.71 ± 23.90
FA 15	30.46 ± 9.07	43.40 ± 44.68	166.84 ± 84.34
FA 30	15.41 ± 4.62	40.34 ± 55.69	147.71 ± 52.42
FA 45	9.84 ± 3.19	24.10 ± 28.22	109.34 ± 55.49
**B**	**Saline (*n*** = **9)**	**50% Vitamin D (*n*** = **9)**	**100 Vitamin D (*n*** = **9)**
FA 3	57.40 ± 30.57	139.10 ± 37.03	98.22 ± 23.18
FA 15	47.96 ± 46.35	43.40 ± 23.80	121.43 ± 36.38
FA 30	38.78 ± 56.76	33.23 ± 32.66	160.66 ± 75.99
FA 45	16.37 ± 25.97	23.20 ± 16.24	108.04 ± 49.11

### T1 values

**Fig. 6:**
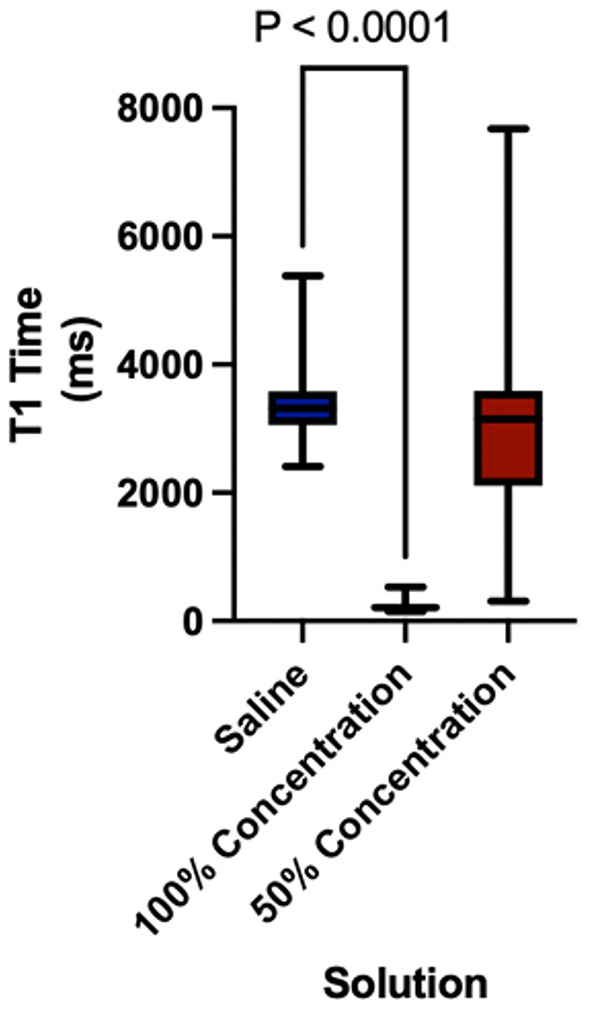
Box plot illustrating T1 relaxation time (ms) for three different solutions: Saline (*n* = 36), 100% concentration (*n* = 32), and 50% concentration (*n* = 32)

T1 values were lower in 100% vitamin D–impregnated catheters compared to the saline control (228 ± 67 ms vs. 3371 ± 493 ms, respectively; *p* < 0.0001), indicating a stronger signal (Fig. 6). T1 values were also lower for 50% vitamin D–impregnated catheters compared to saline (2903 ± 1180 ms vs. 3371 ± 493 ms, respectively; *p* = 0.0108).

## Discussion

This work demonstrates the feasibility of impregnating 3D-printed catheters with vitamin D for passive tracking in MR-guided procedures. 3D printing of the customized catheter design allowed for vitamin D solution to be positioned in the tip of the catheter. MR demonstrated that different isomers of vitamin D produce signals on T1-weighted GRE sequences. The SNRs and CNRs of the three different Vitamin D isomers did not show any significant differences compared to one another. Still, they did show objectively higher SNRs or CNRs compared to catheters with saline or 50% diluted vitamin D. These findings suggest that impregnation with vitamin D may provide visualization and tracking of catheters during MR-guided procedures, which could help address current challenges such as the artifacts seen in coil-based MR procedures [18].

Mean SNRs for 100% cholecalciferol, 100% ergocalciferol, and 100% calcitriol were 138, 139, and 130, respectively. These values were significantly higher than the mean SNR of 30 seen with the control saline catheters (*p* < 0.001). A similar pattern was seen with CNRs for 100% cholecalciferol, 100% ergocalciferol, and 100% calcitriol, which were 135, 137, and 128, respectively, compared to a saline value of 28 (*p* < 0.001 for all comparisons). Catheters with 50% vitamin D solution showed no statistical difference compared to saline in either mean SNR or CNR, highlighting the importance of concentration in signal and contrast. As for the T1 values, 100% vitamin D solution–impregnated catheters were lower, at 228 ± 67 ms, compared to the saline control of 3371 ± 493 ms, indicating significantly stronger signal properties (*p* < 0.0001).

The catheter printed with the proprietary material previously described as MR-visible (High-Temperature RGD525) [14] did not produce a measurable MR signal in our study. The interval between printing and imaging may have contributed to this difference: in our protocol, catheters were imaged approximately 72 h post-printing, following full cleaning and drying. In contrast, the prior study [14] imaged the phantoms shortly after printing and post-processing, which may have preserved transient signal characteristics not present at later time points. Additionally, batch variation in material formulation may have played a role, although this could not be confirmed.

With the successful detection of MR signals in our study, the 3D-printed vitamin D–impregnated catheter holds potential for MR guided procedures. Using this innovation, procedures traditionally performed under computed tomography or fluoroscopy may be performed under MR, reducing the need for ionizing radiation and offering enhanced soft-tissue contrast. Procedures that are traditionally MR-guided may also see an improvement through the elimination of catheters with coils, which produce significant artifacts. These improvements may offer better visibility and safety during MR-guided procedures [3, 19].

This study also highlights the advancement of 3D-printing technology. 3D-printing technologies offer an unparalleled level of customization that allows for the construction of tailored medical devices to meet the specific needs of medical professionals and their patients and can be manufactured to meet real-time demand at relatively low cost [20].

Although the findings are promising, the in vitro nature of this work limits its generalizability to the clinical setting. Additional testing in biological tissues is needed to further assess the strength and performance of these catheters from both a material sciences perspective, as well as providing adequate contrast with surrounding tissues. The 3D printing process itself and thermoplastic welding technique also posed limitations in this study. The observed leakage in a subset of catheters suggests opportunities for optimizing seal integrity post-infusion, given that the vitamin D was manually introduced into a sealed internal chamber rather than infused by soaking or diffusion.

Future in vivo testing is needed to identify how visibility changes in the context of animal models or cadavers. Additional testing further examining the concentrations across isomers of vitamin D could also provide information to optimize the imaging characteristics of the catheter. For additional optimization, different printing materials should be evaluated, and they should be examined for changes in stability and MR visibility over time.

Eventual animal or human studies should be performed to show the safety and efficacy of these catheters in real-world settings, to provide the framework to begin establishing protocols for their use. This is especially important as 3D-printing technology advances find broader applications in medicine, advancing the potential of MR-guided interventions, and ultimately contributing to improved patient outcomes [21, 22].

## Conclusion

This in vitro study demonstrates that a novel 3D-printed catheter design successfully incorporated vitamin D solution in order to facilitate MR visualization. These catheters need to be tested in phantoms, animals, or cadavers to evaluate their integrity and signal characteristics in tissues. The vitamin D solution signal characteristics may have additional applications beyond MR-guided procedures such as improved visualization of medical devices or use in medical phantoms. These findings may help develop catheters suitable for use in MR-guided procedures.

## Data Availability

The datasets used and/or analysed during the current study are available from the corresponding author on reasonable request.

## Abbreviations

MR: Magnetic Resonance
CT: Computed tomography
3D: Three-dimensional
RF: Radiofrequency
PLA: Polylactic acid
FDM: Fused deposition modeling
GRE: Gradient recalled echo
TE: Time to echo
TR: Repetition time
FA: Flip angle
SNR: Signal-to-noise ratio
CNR: Contrast-to-noise ratio
ms: Milliseconds
